# A Cascaded Neural Network for Robust Phase-Only Beamforming Under Covariance Matrix Mismatch

**DOI:** 10.3390/s26134077

**Published:** 2026-06-26

**Authors:** Zhonghui Zhao, Zhaosheng Yu, Yao Li, Yan Yang, Zhuopeng Wang, Qiang Liu

**Affiliations:** College of Electronic and Information Engineering, Shandong University of Science and Technology, Qingdao 266590, China; zhaoshengy@sdust.edu.cn (Z.Y.); liyao@sdust.edu.cn (Y.L.); yangyan@sdust.edu.cn (Y.Y.); wzhuopeng1@sdust.edu.cn (Z.W.); qiangliu@sdust.edu.cn (Q.L.)

**Keywords:** covariance-mismatch-tolerant phase-only beamforming, covariance matrix mismatch, denoising autoencoder, residual network

## Abstract

**Highlights:**

**What are the main findings?**

- A cascaded DAE–ResNet neural network is proposed for covariance-mismatchtolerant phase-only beamforming.
- The DAE mitigates covariance matrix mismatch, while the ResNet efficiently emulates phase-only excitation solutions.

**What are the implications of the main findings?**

- The proposed framework improves output SINR robustness under limited-snapshot and low-SNR covariance-estimation conditions.
- The method reduces online computational cost by replacing repeated iterative optimization with neural network inference.

**Abstract:**

This paper presents a cascaded neural network framework for phase-only beamforming under covariance matrix mismatch. The proposed architecture combines a denoising autoencoder (DAE) with a residual network (ResNet) to address performance degradation caused by finite-snapshot covariance estimation errors and signal-of-interest contamination. The DAE reconstructs an ideal covariance representation from mismatched covariance inputs and provides compact covariance features for subsequent phase prediction. The ResNet then maps the denoised covariance features to phase-only excitation vectors, thereby avoiding repeated online optimization. Unlike conventional robust adaptive beamforming methods that rely on explicit uncertainty modeling or iterative covariance reconstruction, the proposed framework separates covariance feature denoising from phase excitation emulation in a data-driven manner. Numerical results demonstrate that the cascaded network improves covariance-mismatch tolerance and achieves competitive output SINR performance under limited-snapshot and noisy covariance-estimation conditions.

## 1. Introduction

Adaptive beamforming adjusts the complex weights of antenna elements to enhance the signal of interest while suppressing interference. It has been widely used in wireless communication and radar systems [1]. In many practical arrays, hardware cost and power consumption motivate the use of phase-only control, where pattern synthesis is achieved by adjusting only the phase terms. This architecture avoids variable amplitude weighting and simplifies the feeding network. However, the associated design problem is nonlinear and nonconvex, which makes efficient phase-only beamformer synthesis challenging.

Several approaches have been developed for phase-only beamforming, including iterative numerical methods [2,3], evolutionary optimization [4,5], and convex optimization-based formulations [6,7]. These methods can provide effective phase excitations under various pattern constraints. However, these methods usually require iterative optimization or parameter search for each new array scenario, and the computational burden increases with the array size and the number of constraints.

Robust adaptive beamforming has been extensively investigated to mitigate performance degradation caused by model mismatch. Representative model-based techniques include eigenspace projection, robust Capon beamforming, diagonal loading, steering-vector estimation, and covariance matrix reconstruction. These approaches improve robustness by refining the steering vector model, suppressing signal-of-interest contamination, or reconstructing the interference-plus-noise covariance matrix [8,9,10]. Recent covariance-reconstruction methods further incorporate robust Capon principles and diagonal loading strategies [11,12]. Other studies have considered Gauss–Legendre quadrature-based reconstruction and quasi-signal-subspace estimation [13,14]. In addition, complex-valued convolutional neural networks have been explored for robust sensor-array beamforming [15]. These studies provide important references for model-driven and data-driven robustness enhancement. Nevertheless, most model-based robust adaptive beamformers are formulated for fully complex-valued adaptive weights and require matrix decomposition, uncertainty modeling, covariance reconstruction, or iterative optimization. Existing deep-learning-based robust beamformers improve online prediction efficiency, but they generally do not explicitly decouple covariance mismatch mitigation from phase-only excitation emulation.

Neural networks (NNs) have recently been used as data-driven surrogates for beamforming optimization [16,17]. Back-propagation networks and radial basis function networks have been applied to phase-only mainlobe steering and nulling [18,19]. More recent neural beamforming frameworks have addressed interference rejection, pattern synthesis, and hybrid beamforming problems [20,21,22]. These works indicate that neural networks can approximate complex beamforming mappings and reduce online computational cost after being trained offline. However, most existing neural beamforming methods directly learn the mapping from array information to beamforming outputs, without separately modeling the covariance mismatch mitigation process.

In practical antenna arrays, short data records, signal-of-interest contamination, and steering-vector uncertainty may lead to covariance matrix mismatch and beamforming performance loss [23]. It is therefore necessary to develop phase-only beamforming methods that can tolerate covariance mismatch under finite-snapshot and noisy covariance-estimation conditions.

Motivated by these considerations, this paper proposes a covariance-mismatch-tolerant phase-only beamforming framework based on a cascaded DAE–ResNet architecture. A phase-only beamforming model with a sidelobe-level constraint is first formulated. Instead of directly predicting phase excitations from mismatched covariance matrices, the proposed framework separates covariance feature denoising from phase excitation emulation. The DAE reconstructs a compact covariance representation and extracts mismatch-tolerant features [24,25]. The ResNet then predicts the corresponding phase-only excitation vector using the denoised covariance features and the desired steering vector. The residual structure is adopted to improve training stability [26]. Simulation results demonstrate that the proposed cascaded network improves phase-only beamforming performance under covariance matrix mismatch caused by finite snapshots and noisy covariance estimation.

The remainder of this paper is organized as follows. Section 2 formulates the phase-only array beamforming problem. Section 3 describes the proposed cascaded DAE–ResNet framework. Section 4 presents the numerical results and discussion. Section 5 concludes the paper.

## 2. Problem Formulation

Consider an array consisting of *M* elements receiving narrowband signals from multiple independent sources. The received signal vector at time index *k* is expressed as

(1) $$x\left(k\right)=s\left(k\right)a\left(\theta_{0}\right)+v\left(k\right),$$

where s(k) denotes the waveform of the signal of interest (SOI), $a(\theta_{0})$ is the steering vector corresponding to the desired direction $\theta_{0}$, and v(k) represents the interference-plus-noise component.

The minimum variance distortionless response (MVDR) beamformer aims to minimize the output interference-plus-noise power while maintaining a distortionless response toward the SOI [27]:

(2) $$\begin{array}{cc} \min_{w}\; & w^{H}R_{i+n}w\\ s.t.\; & w^{H}a\left(\theta_{0}\right)=1, \end{array}$$

where w denotes the beamforming weight vector and $R_{i+n}$ is the interference-plus-noise covariance matrix.

For phase-only beamforming, the amplitude excitations are fixed, and only the phase excitations are adjusted. Under this constraint, perfect distortionless response cannot generally be guaranteed, and mainlobe gain distortion as well as sidelobe level elevation may occur [6]. To ensure a minimum acceptable gain in the desired direction while suppressing sidelobe radiation, the phase-only beamforming problem is formulated as

(3) $$\begin{array}{cc} \min_{w}\; & w^{H}R_{i+n}w\\ s.t.\; & \left|w^{H}a\left(\theta_{0}\right)\right|\geq \delta ,\\ \; & \left|w^{H}a\left(\theta_{s}\right)\right|\leq \eta ,\;\theta_{s}\in \Omega_{s},\\ \; & w=\alpha ⊙e^{j\phi }, \end{array}$$

where δ and η denote the gain constraint in the desired direction and the maximum allowable sidelobe level, respectively, $\Omega_{s}$ represents the sidelobe angular region, α is the fixed amplitude excitation vector, ϕ contains the phase excitation variables, and ⊙ denotes the Hadamard product.

When only the phase variables ϕ are optimized, the above problem is NP-hard. Near-optimal phase solutions can be obtained using semidefinite relaxation (SDR) techniques [28]. In this work, the direct iterative rank refinement (DIRR) algorithm is adopted to obtain the phase excitations [29].

For each generated array scenario, the DIRR-based phase-only beamformer is used as the offline label-generation solver. Given the ideal interference-plus-noise covariance matrix and the desired steering vector, the optimization problem in (3) is solved by the DIRR algorithm combined with a golden-section search over δ. The resulting phase vector is denoted as $\phi^{★}$ and is used as the supervised target for the ResNet-based phase excitation emulation network.

In practice, the covariance matrix $R_{i+n}$ is unknown and is typically approximated by the sample covariance matrix

(4) $$\hat{R}=\frac{1}{N}\sum_{n=1}^{N}x\left(n\right)x^{H}\left(n\right),$$

where x(n) denotes the received snapshot vector and *N* is the number of snapshots.

Moreover, it has been reported that the output SINR of the phase-only beamformer is sensitive to the choice of the constraint parameter δ [30,31]. To determine an appropriate value of δ, a golden-section search is employed. Algorithm 1 summarizes the search procedure. At each iteration, two candidate values of δ are evaluated by the DIRR solver, and the search interval is updated according to the corresponding output SINR until the stopping tolerance is satisfied.
**Algorithm 1:** The procedure of the phase-only beamformer**Input:**     $\delta_{l}$, $\delta_{u}$, and $\hat{R}$ **        1:     **Set $\delta_{1}=\delta_{u}-\left(\delta_{u}-\delta_{l}\right)/1.618$ and $\delta_{2}=\delta_{l}+\left(\delta_{u}-\delta_{l}\right)/1.618$ **        2:     **Use the DIRR algorithm to solve (3) with $\delta =\delta_{1}$ and $\delta =\delta_{2}$, respectively **        3:     **Compute the corresponding $\mathrm{SINR}(\delta_{1})$ and $\mathrm{SINR}(\delta_{2})$ **        4:     if** $\mathrm{SINR}\left(\delta_{1}\right)<\mathrm{SINR}\left(\delta_{2}\right)$                     $\delta_{u}\leftarrow \delta_{2}$, $\delta_{2}\leftarrow \delta_{1}$, $\delta_{1}\leftarrow \delta_{u}-\left(\delta_{u}-\delta_{l}\right)/1.618$                     **else**                     $\delta_{l}\leftarrow \delta_{1}$, $\delta_{1}\leftarrow \delta_{2}$, $\delta_{2}\leftarrow \delta_{l}+\left(\delta_{u}-\delta_{l}\right)/1.618$ **                end if** **        5:     if** $\delta_{u}-\delta_{l}<0.01$, then stop; otherwise go to step 2 **        6:     Output:** the corresponding phase excitations

The phase excitation vector returned by Algorithm 1 is used as the conventional phase-only solution and as the supervised label for training the phase excitation emulation network.

## 3. Cascaded NN-Based Phase-Only Beamforming Framework

The proposed framework improves phase-only beamforming under covariance matrix mismatch by separating covariance feature denoising from phase excitation emulation. Instead of directly mapping mismatched sample covariance matrices to phase solutions, the framework first uses a denoising autoencoder (DAE) to extract a compact covariance representation. The denoised features are then supplied to a residual network (ResNet), which emulates the nonlinear mapping from covariance information to phase-only excitations. The DAE module, the phase excitation emulation network, and the training procedure are described below.

### 3.1. DAE for Sample Covariance Matrix Reconstruction

In practical array processing, the sample covariance matrix may differ significantly from the ideal interference-plus-noise covariance matrix, especially when only a limited number of snapshots is available or when the covariance estimate is contaminated by the signal of interest. This mismatch may noticeably degrade phase-only beamforming performance. Covariance reconstruction and refinement have therefore been widely used to mitigate its impact [8,9].

**Figure 1:**
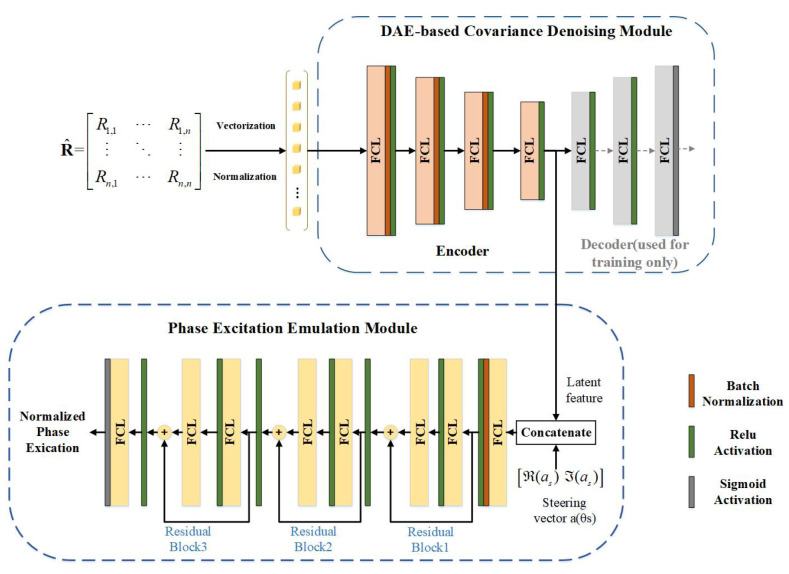
Cascaded neural network framework for covariance-mismatch-tolerant phase-only beamforming.

Motivated by this idea, the proposed framework introduces a DAE to learn a nonlinear mapping from mismatched sample covariance representations to their ideal counterparts. The sample covariance matrix is used as the DAE input, and the corresponding ideal covariance matrix is used as the reconstruction target. The DAE suppresses mismatch-induced perturbations while retaining the spatial information required for beamforming. The overall cascaded architecture is shown in Figure 1.

As shown in Figure 1, the DAE consists of an encoder and a decoder [32]. The encoder compresses the high-dimensional covariance representation into a lower-dimensional latent feature that preserves the principal spatial structure of the array environment. The decoder reconstructs the covariance representation from this latent feature. Fully connected layers, batch normalization, and ReLU activation functions are used in the encoder, while the decoder adopts a symmetric fully connected structure. A sigmoid activation function is used in the final decoder layer to keep the reconstructed output within the normalized range.

Since the DAE uses real-valued inputs, the complex covariance matrix is first reformulated and normalized following [33]. Specifically, the upper triangular elements of the covariance matrix are vectorized as

(5) $$\bar{r}={\left[R_{1,1},R_{1,2},\ldots ,R_{1,M},R_{2,2},\ldots ,R_{M,M}\right]}^{T}\in C^{M(M+1)/2},$$

where $R_{m_{1},m_{2}}$ denotes the $\left(m_{1},m_{2}\right)$ th entry of the covariance matrix. The real-valued input vector is then constructed as

(6) $$\hat{r}=\frac{{\left[ℜ\left\{\bar{r}\right\},\;ℑ\left\{\bar{r}\right\}\right]}^{T}}{∥\bar{r}{∥}_{F}},$$

where ℜ{·} and ℑ{·} denote the real and imaginary parts, respectively. For the considered 10-element array, the upper-triangular representation leads to a 110-dimensional real-valued input after separating the real and imaginary parts. Min–max normalization is then applied to scale the elements of $\hat{r}$ into the interval [0,1].

The DAE is trained by minimizing the mean squared error (MSE) between the normalized sample covariance representation and the corresponding normalized ideal covariance representation. Only the encoder output is used in the subsequent phase excitation emulation stage, because the latent feature contains the spatial information needed for phase prediction without requiring explicit covariance reconstruction during inference.

### 3.2. ResNet for Phase Excitation Emulation

The ResNet module emulates the phase excitations produced by an optimization-based phase-only beamformer. Rather than mapping the sample covariance matrix directly to phase solutions, this module learns a stable nonlinear mapping from the denoised covariance feature to the phase excitation vector.

The phase-only beamforming problem is highly nonlinear, and the phase solutions obtained from optimization-based solvers can be sensitive to covariance perturbations and numerical approximation errors. A sufficiently expressive network is therefore needed to capture the input–output relationship while maintaining stable training. To this end, a residual architecture is adopted. The identity skip connections in ResNet help alleviate gradient degradation and improve the trainability of deeper fully connected networks [26].

As shown in Figure 1, the ResNet input is formed by concatenating the latent covariance feature extracted by the DAE with the real and imaginary parts of the normalized steering vector:

(7) $$[r_{\mathrm{code}},\;ℜ\left({\hat{a}}_{s}\right),\;ℑ\left({\hat{a}}_{s}\right)].$$

Here, $r_{\mathrm{code}}$ denotes the encoded covariance feature, and ${\hat{a}}_{s}$ denotes the normalized steering vector corresponding to the desired direction.

The Huber loss is used to improve the stability of phase excitation learning [34]. Compared with a pure squared-error loss, the Huber loss reduces the influence of occasional large deviations in the training targets while retaining efficient convergence for small prediction errors:

(8) $$H_{e}\left(t\right)=\left\{\begin{array}{cc} \frac{t^{2}}{2}, & |t|<c,\\ c|t|-\frac{c^{2}}{2}, & |t|\geq c, \end{array}\right.$$

where *t* is the prediction error and *c* is the transition threshold. This loss function is suitable for phase-emulation training because the phase labels generated by relaxation-based optimization may contain small numerical deviations from the ideal rank-one solution.

### 3.3. Cascaded NN Training Strategy for Phase-Only Beamforming

The proposed network is trained in two stages. In the first stage, sample covariance matrices are generated under different snapshot numbers, signal-to-noise ratios, and noise realizations. The corresponding ideal interference-plus-noise covariance matrices are used as reconstruction targets. This stage trains the DAE to extract covariance features that are less sensitive to finite-snapshot and noise-induced mismatch.

In the second stage, supervised training pairs are generated for phase excitation emulation. For each array scenario, the ideal covariance matrix $R_{i+n}$, the desired steering vector $a(\theta_{0})$, and the optimized phase excitation vector $\phi^{★}$ form one training sample. The target phase vector $\phi^{★}$ is obtained by solving the phase-only beamforming problem in (3) using the DIRR-based procedure summarized in Algorithm 1. The optimized phase values are wrapped into (−π,π] and normalized as

(9) $$y_{\phi }=\frac{\phi^{★}+\pi }{2\pi },$$

where $y_{\phi }$ is the normalized phase label.

The ideal covariance matrix is processed by the trained DAE to obtain $r_{\mathrm{code}}$. This latent feature is concatenated with the real and imaginary parts of the normalized steering vector and used as the ResNet input. The ResNet is trained to approximate

(10) $$f_{\mathrm{ResNet}}:\left[r_{\mathrm{code}},ℜ\left({\hat{a}}_{s}\right),ℑ\left({\hat{a}}_{s}\right)\right]\to y_{\phi }.$$

After training, the predicted normalized phase vector is transformed back to the physical phase excitation by

(11) $$\hat{\phi }=2\pi {\hat{y}}_{\phi }-\pi .$$

During online inference, the received snapshots are first used to estimate the sample covariance matrix. The trained DAE then extracts a covariance feature, and the trained ResNet directly predicts the corresponding phase-only excitation vector. Thus, the iterative DIRR optimization is used only for offline label generation and is not repeated during online inference.

## 4. Simulation Results and Discussion

This section evaluates the proposed cascaded neural beamformer under covariance matrix mismatch. The simulations assess covariance reconstruction accuracy, phase excitation emulation, output SINR performance, and computational efficiency.

### 4.1. Simulation Setup

A 10-element uniform linear array with half-wavelength inter-element spacing is considered. In the phase-only beamforming model in (3), the initial amplitude excitation follows a −18 dB Taylor taper, and the sidelobe level constraint is set to −15 dB. The desired direction is randomly sampled from [−10°,10°], while the interference directions are sampled from the sidelobe region within [−60°,60°]. The maximum number of interference sources is set to three.

### 4.2. Performance of Covariance Matrix Reconstruction

The DAE is first evaluated for its ability to mitigate covariance mismatch. The training set contains 300,000 covariance matrix samples generated under randomly distributed signal-to-noise ratios from 10 dB to 25 dB and snapshot numbers from 10 to 300. The data are divided into training and validation subsets with ratios of 85% and 15%, respectively. The Adam optimizer [35,36] is used with a batch size of 1024 and a maximum of 400 epochs. The initial learning rate is 0.001 and is reduced by a factor of 0.7 when the validation loss reaches a plateau with a patience of 50 epochs. The DAE architecture is 110-150-90-60-90-150-90-110, which is consistent with the upper-triangular covariance input representation.

The reconstruction accuracy is measured by the normalized Frobenius distance:

(12) $$D=\frac{{∥R_{\mathrm{ideal}}-\hat{R}∥}_{F}}{{∥R_{\mathrm{ideal}}∥}_{F}},$$

where $R_{\mathrm{ideal}}$ and $\hat{R}$ denote the ideal and reconstructed covariance matrices, respectively.

**Figure 2:**
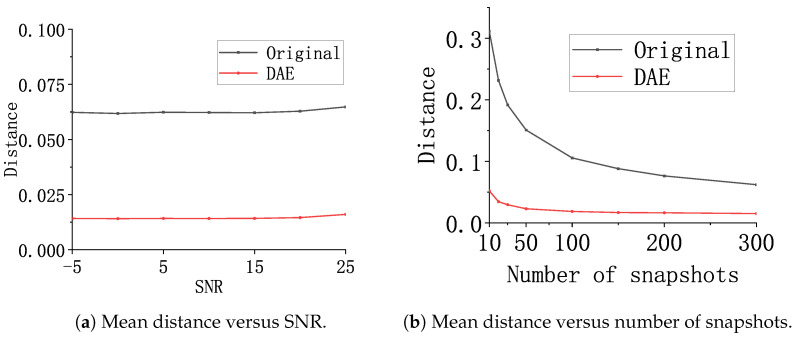
Reconstruction performance of the DAE.

Figure 2 shows the average reconstruction distance for 2000 unseen test cases. The sample covariance matrices deviate noticeably from the ideal covariance matrices, especially under low-SNR or limited-snapshot conditions. In comparison, the DAE-reconstructed covariance representations yield lower reconstruction errors over the tested SNR and snapshot ranges. These results confirm that the DAE provides effective covariance feature denoising before phase excitation emulation.

### 4.3. Training Performance of Phase Excitation Emulation Network

**Figure 3:**
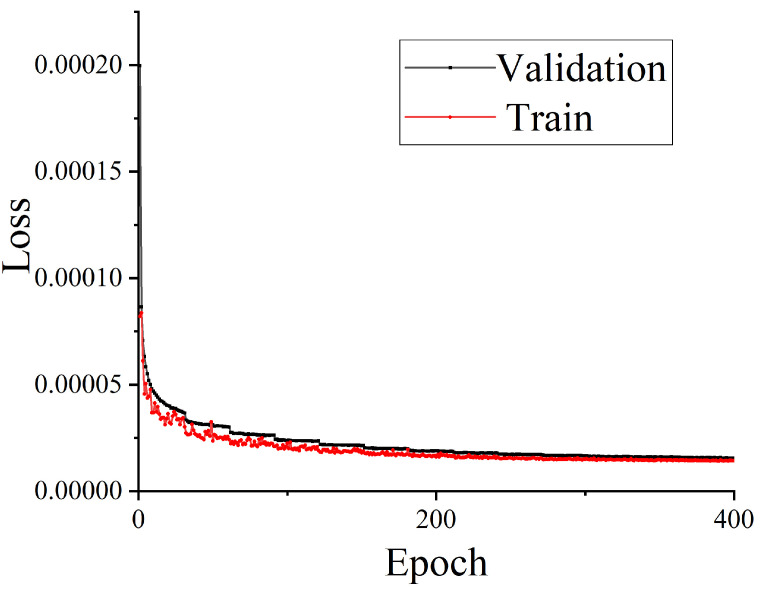
Training and validation loss versus epochs.

The ResNet-based phase excitation emulation network is trained using 20,000 input–output pairs. The target phase excitations are generated by the conventional phase-only beamformer. The data are split into training and validation sets with ratios of 85% and 15%, respectively. All hidden fully connected layers contain 128 neurons. The hyperparameters are the same as those used for the DAE, except that the batch size is set to 32. Figure 3 shows the training and validation loss curves. The validation loss converges to $4.71\times 10^{-6}$ after 400 epochs, indicating that the ResNet accurately approximates the phase excitation labels generated by the optimization-based beamformer.

### 4.4. Output SINR Performance Analysis

The proposed cascaded neural beamformer is compared with three reference methods: the ideal phase-only beamformer using the true covariance matrix, the conventional phase-only beamformer using sample covariance matrices, and the ResNet-based beamformer without the DAE module. The desired signal is assumed to arrive from 10°, while three interference signals arrive from −48°, −41°, and −28°. The interference-to-noise ratios are set to 30 dB. For each setting, 50 Monte Carlo trials are performed.

**Figure 4:**
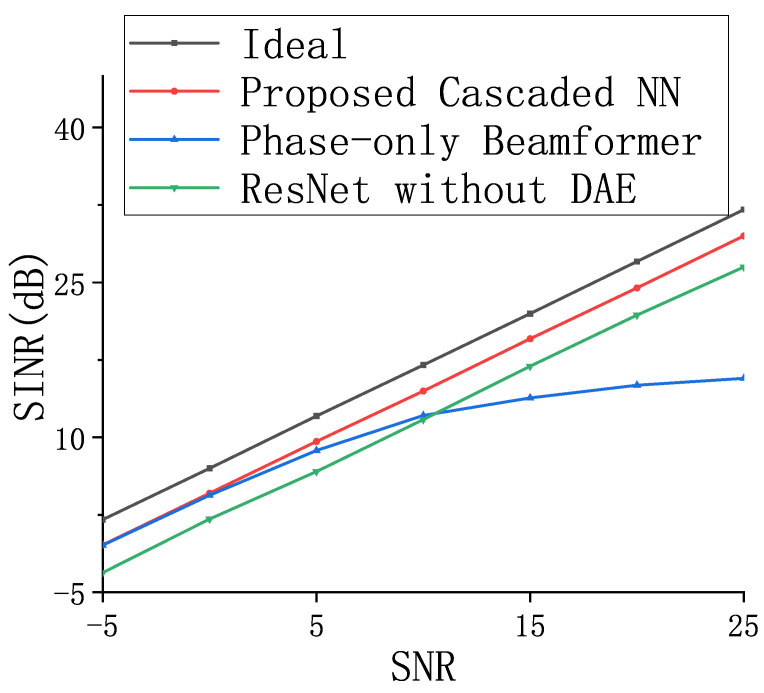
Output SINR versus SNR.

Figure 4 shows the output SINR versus input SNR when the number of snapshots is fixed at 150. The proposed cascaded network achieves SINR performance close to that of the ideal beamformer across the tested SNR range. The conventional phase-only beamformer suffers from noticeable degradation at high SNR because the sample covariance mismatch becomes more influential. The ResNet-only model also shows limited mismatch tolerance, which confirms the benefit of the DAE-based covariance feature denoising stage.

**Figure 5:**
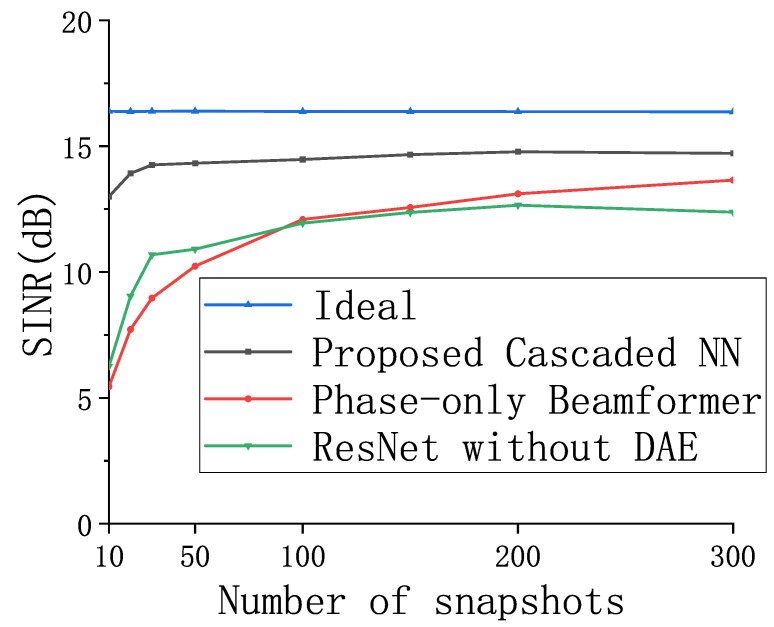
Output SINR versus the number of snapshots.

Figure 5 shows the output SINR versus the number of snapshots at an SNR of 10 dB. All methods degrade when the number of snapshots is small. However, the proposed cascaded neural beamformer consistently outperforms the conventional phase-only beamformer and the ResNet-only model. When the number of snapshots is below 50, the proposed method provides an SINR improvement of approximately 5 dB over the conventional phase-only beamformer.

### 4.5. Computational Complexity and Implementation Discussion

The proposed framework includes an offline training stage and an online inference stage. During offline training, the DAE is trained using 300,000 covariance matrix samples, and the ResNet is trained using 20,000 input–output phase excitation pairs. This training process is performed once and is not required during online beamforming. During online inference, the received snapshots are used to estimate the sample covariance matrix, the trained DAE extracts the latent covariance feature, and the trained ResNet predicts the phase-only excitation vector. Thus, the DIRR-based optimization and golden-section search are used only for offline label generation.

For the considered 10-element array, the DAE uses a 110-150-90-60-90-150-90-110 fully connected structure, corresponding to the upper-triangular covariance input representation. The saved DAE used during online inference has a model size of 249 KB, and the saved ResNet-based phase excitation emulation network has a model size of 477 KB. These model sizes indicate that both neural modules are lightweight for online deployment.

The runtime was measured on a desktop workstation with an Intel i7-8700 CPU and 16 GB RAM. The neural network models were implemented and tested in an Anaconda-based Python 3.7 environment. The testing time of the DAE was approximately 0.11 s, and that of the ResNet phase excitation emulation network was approximately 0.08 s. The total neural network inference time of the cascaded DAE–ResNet framework was therefore approximately 0.19 s. For comparison, the conventional phase-only nulling method implemented using an iterative convex-optimization solver required approximately 5–8 s, depending on the number of iterations. These results indicate that the proposed framework reduces the online computational cost by replacing repeated optimization with neural network inference.

## 5. Conclusions

This paper proposed a cascaded DAE–ResNet framework for phase-only beamforming under covariance matrix mismatch. The DAE reconstructs a compact covariance representation from mismatched sample covariance inputs, while the ResNet emulates the corresponding phase-only excitation vector. By separating covariance feature denoising from phase excitation emulation, the proposed method improves covariance-mismatch tolerance under limited-snapshot and low-SNR conditions. Numerical results show that the cascaded network achieves output SINR performance close to that of the ideal beamformer and consistently outperforms both the conventional phase-only beamformer and the ResNet-only model. The measured inference time on the adopted workstation further indicates lower online computational cost than the iterative convex-optimization implementation. Future work will further investigate the robustness of the proposed method against various model errors, including look-direction deviations, mutual coupling effects, channel mismatch, gain and phase perturbations, as well as antenna position errors.

## Data Availability

Data will be available upon reasonable request.
